# Concentrations of Salivary Cortisol in Victims of Intimate Partner Violence According to the CIRCORT Database

**DOI:** 10.3390/ijerph182010819

**Published:** 2021-10-14

**Authors:** Sarai Mata-Gil, Antonio Sánchez-Cabaco, Jerónimo Del Moral-Martínez, Antonio Seisdedos-Benito, Ulf Lundberg

**Affiliations:** 1 Department of Psychology, Campus Universitario, University of Extremadura, 06006 Badajoz, Spain; 2 Faculty of Psychology Pontifical, University of Salamanca (UPSA), 37002 Salamanca, Spain; asanchezca@upsa.es; 3 Faculty of Science, Campus Universitario, University of Extremadura (UEX), 06006 Badajoz, Spain; jeronimodelmoral@yahoo.es; 4 Faculty of Psychology, Campus Ciudad Jardín, University of Salamanca (USAL), 37005 Salamanca, Spain; aima6d2@usal.es; 5 Department of Psychology, Biology Division, Stockholm University, SE-106 91 Stockholm, Sweden; ul@psychology.su.se

**Keywords:** woman, cortisol saliva, intimate partner violence, cross-sectional survey

## Abstract

This work analyzes the different levels of salivary cortisol in women from the southwest of Spain that were victims of intimate partner violence (IPV) with respect to a control group, assessing for the first time the different concentrations obtained in relation to a worldwide reference standard provided by the CIRCORT meta-global cortisol database. The clinical sample (*N* = 24) and the control group (*N* = 25) had an average of 39.12 years (*SD* = 12.31) and 39.52 years (*SD* = 11.74), respectively. Cortisol awakening response (CAR) was determined by defining the area under the curve (AUCi). There were no differences between the CAR data of the two populations *F* (1, 141) = 1.690, *p* = 0.196, but there was a highly significant difference in the three sampling days, where the clinical sample exceeded the cortisol levels of the CIRCORT database in the evening as compared to the control group (*p* = 0.004, *p* = 0.001 and *p* = 0.000). Salivary cortisol concentration samples taken in the evening were significantly higher than those standardized in the CIRCORT database, from the women victims of IPV as compared to the control group, showing its usefulness as an effective supportive tool for problems such as those triggered by IPV.

## 1. Introduction

Intimate partner violence (IPV) is defined by the Centers for Disease Control and Prevention as a type of violence that “includes physical violence, sexual violence, stalking and psychological aggression (including coercive tactics) by a current or former intimate partner (i.e., spouse, boyfriend/girlfriend, dating partner, or ongoing sexual partner)” [1].

Exposure to IPV is associated with psychological stress and stress-related endocrine and immune-inflammatory dysregulations [2,3], although some works have exposed that classification guides, such as ICD-11, do not yet adequately capture it [4].

Situations of stress, such as those triggered by IPV, are biologically regulated by the hypothalamic–pituitary–adrenal axis (HPA) [5,6] where the hypothalamus activates in the pituitary gland the release of hormones, such as adenocorticotropin (ACTH), in the blood. This in turn stimulates the suprarenal cortex to release cortisol in the blood, a steroid hormone with a wide spectrum of activity in the body, which can lead to subsequent effects in the brain [7]. This cascade of physiological responses regulated by the HPA can be altered when confronted with psychosomatic and psychiatric disorders such as anxiety, depression and the post-traumatic stress disorder (PTSD) [8], which can be triggered in situations of IPV [9,10].

The rapid increase in cortisol levels upon awakening, known as the cortisol awakening response (CAR), has been established as a promising biomarker in psychoneuroendocrinology [11] and is an indicator of the activity of the HPA [12], providing that its regulation and significance are previously defined in healthy individuals [13]. In addition, this response should be considered as a flexible parameter with respect to different factors, rather than as a specific measurement [14]. The use of CAR has become increasingly prevalent, as initiatives have been made to standardize the protocols for determining the response [15] Furthermore, its effectiveness is conditioned by the fact that samples can be taken on two or more days, since single-day sample collection is subject to a wide variety of environmental factors, which make drawing precise conclusions often difficult [16].

Despite the evidence of altered cortisol levels in women with experiences of abuse [17,18,19], women victims of IPV [20], as well as PTSD [21], anxiety, depression and problems with self-esteem [22], there are studies where the differences in cortisol levels are only associated with the chronicity of the abuse of the victims [23], where the physical stressor is the only variable responsible for altering cortisol levels [24].

Deviations in cortisol levels have been standardized in a study in which different cortisol levels have been analyzed in the meta-global analysis CIRCORT. These different cortisol levels have been grouped according to sex and age and distributes them in percentiles [25], generating a relevant tool for identifying altered cortisol levels outside normal percentiles.

In the present study, the salivary cortisol data from a population of women victims of IPV is compared with that of a control population, assessing the different concentrations obtained, for the first time, in relation to the CIRCORT global cortisol database, with the aim of advancing the effective diagnosis of the negative health consequences in female victims of IPV, which to date is still limited [26].

## 2. Materials and Methods

### 2.1. Participants

The participants in this study included 50 women of which 25 were victims of IPV, forming the clinical sample. The other 25 women formed the non-clinical sample (non-victims of IPV women), and all women in both groups were selected from the southwest of Spain in the autonomous communities of Castile, Leon and Extremadura.

The clinical sample (women victims of IPV) resided in institutionalized shelters/homes. The average age of the women was 39 years ($\bar{X}$= 39.12, *SD* = 12.31) and their marital status was 52% single, separated and/or with boyfriends (13 women), 28% (7 women) married and 20% (5 women) in the process of separation. The average number of children was 2 ($\bar{X}$= 2.08, *SD* = 1.412). Seventy-six percent of the women had Spanish nationality (19 women), 16% (4 women) were from Latin America and 8% (2 women) had other nationalities. Sixteen percent of the women (4) presented a disability/incapacity, while the rest, 84% (21 women), did not.

The educational levels included: 60% of the women had a primary level of education (15 women), 24% had completed secondary studies/professional training (6 women), 12% (3 women) held university degrees and 4% (1 woman) had not attended school. Eighty-eight percent of the women had previous work experience (22), compared to the 12% (3 women) who had never worked. With regard to the type of abuse, some women had been exposed to combinations of different forms of violence, which explains the high percentage, 80% was physical abuse (20 women), 96% was psychological abuse (24 women) and 40% (10 women) was sexual abuse; 12% (3 women) of the women did not respond due to embarrassment. Fifty-six percent of the women (14 women) were raped and forced to have sexual relations and referred to the relations as “compulsory”. In addition, 56% (14 women) reported abuse during pregnancy. Three of the women were unable to answer the questions due to fear and emotional blockages and had a history of abuse of an average of 13 years ($\bar{X}$ = 13.22). Additionally, all women in the clinical sample had to have met the following inclusion criteria: be over 18 years of age, have filed a police report against the alleged abuser, fulfilled the compliance requirements for their acceptance in a specialized center, and knowledge of the Spanish language. The exclusion criteria included: the presence of an intellectual disability that prevented the understanding of the informed consent to participate in the study, a psychiatric illness (psychosis), addictions (alcoholism and drug addiction) and in cases where the individual was in the process of receiving psychotherapy.

The other 25 women in the non-clinical sample (non-victims of IPV women) had an average age of 39 years ($\bar{X}$ = 39.52, *SD* = 11.74). Forty-eight percent of the women (12 women) were single, separated and/or with a boyfriend, 52% (13 women) were married, and the average number of children was 2 ($\bar{X}$ = 1.80 and *SD* = 1.55), Eighty-eight percent had Spanish nationality (22 women), 8% were Latin American (2 women) and 4% (1 woman) had another nationality. Sixteen percent had a disability/incapacity (4), as compared to the 84% (21 women) who did not. The educational levels included: 40% of the women had a primary level of education (10 women), 32% had secondary studies/professional training (8 women) and 28% (7 women) held university degrees. Seventy-six percent had previous work experience (19 women) compared to the 24% (6 women) who did not. However, the women in this non-clinical sample did not have the same sociocultural characteristics as the clinical sample, since 64% (16 women) had been selected from Badajoz as volunteering participants from the Association of Centers for the Culture and Promotion of Women. The remaining 36% (9 women) were women volunteers from Badajoz that did not belong to any association and were recruited because they had similar sociodemographic and cultural situations as the clinical sample.

Similar to the clinical sample, the non-clinical sample had to fulfill certain inclusion criteria which were: knowledge of the Spanish language, to be at least 18 years of age and have some sociodemographic similarities with the selected clinical sample. The exclusion criteria were again the same as the non-clinical sample: the presence of an intellectual disability that prevented the understanding of the informed consent to participate in the study, a psychiatric illness (psychosis), addictions (alcoholism and drug addiction) and in cases where the individual was in the process of receiving psychotherapy.

### 2.2. Procedure

In order to develop this research using a sample of women victims of IPV, it was necessary to respect the protocol of institutional protection, owing to the critical period suffered by the victims and the security and law enforcement that ensured maximum confidentiality and avoided different risk factors [27].

The work with the clinical sample (*N* = 25) involved transporting the women such that personal interviews could take place with the management and technical team (psychiatrist, psychologist, social worker and caregivers) of each of the participating shelters.

The study consisted of establishing a coordinated protocol with the psychologist and social worker of each shelter/shelter apartment such that at the time of a victim’s admission in the center (respecting the adaptation process of each shelter for a few days) they were informed of the research study and procedure that was going to be followed. This occurred after respecting the adaptation process for several days and in the cases where the women had agreed to participate. Subsequently, the psychologist participating in the study was contacted and immediately travelled to the relevant shelter. A minimum of 2–5 sessions (90 min per session) occurred with each victim in order to guarantee adequate adherence to the study, at which time the instructions regarding participation and the collection of the salivary sample were explained. In addition, personal interviews occurred for collecting the information regarding the different variables (personal, socio-economic, social/family, social context, habits, motivations and social participation). Personal interviews were conducted for both the clinical and non-clinical samples. In addition, information was collected regarding variables related to the stressor, such as the relationship between the victim and the person the complaint was filed against (the alleged abuser), psychosomatic abuse, and any legal, psychological and/or social assistance. Psychological tests—for detecting anxiety, post-traumatic stress disorder (PTSD), alexithymia, low self-esteem, depression were developed for both the clinical and non-clinical samples selected.

In order for all of the information to be processed, a questionnaire-based database was designed. In addition, the participants were asked to sign a participation and contribution to the study agreement that was in accordance with the Declaration of Helsinki, as well as informed and voluntary consent, and the anonymous transfer of their data for research purposes. Each woman was also informed about the protocol for recording the interviews, which would be used for carrying out an exhaustive collection of information for each participant.

In addition, each woman was given a set of material properly identified with a file number, two telephone contacts and the direct email address of the psychologist who was attending them in the case of any questions or the need for clarification. The women were also given a schedule that outlined when they had to collect their saliva, indicating the 1st sample, immediately before getting out of bed (S1), the 2nd sample, 30 min after getting up (S2), and the 3rd sample, in the evening, after 660 min (11 h) from the first sample collection (S3) for three days. The CAR (Cortisol Awakening Response) was determined from the salivary cortisol samples obtained at the time of getting up and then 30 min later, defining the area under the curve with respect to the increase (AUCi) of the data. To avoid errors, those samples that were obtained with a delay equal to or greater than 15 min after getting up were eliminated from the study, since delays greater than 15 min subsequently reduced CAR values. [16].

An instruction protocol was also provided, (as a reminder) explaining how to chew the cotton-like chewing gum and introduce it into a hermetically sealed capsule in a saliva collection tube (Salivette, Sarstedt, Nümbrecht, Germany). This tube would then immediately be deposited in the airtight bag provided, identified with the participant’s file number and finally placed in the freezer.

The women were also told that in the morning they had to perform the tests before eating, smoking or brushing their teeth; therefore, food intake or smoking was absolutely prohibited before sample collection. Likewise, for the evening sample collection, the participants could not eat, smoke or drink, except for water, or brush their teeth half an hour before taking the sample. In addition, the caretaking staff of each shelter informed the women of how to effectively carry out the process, where information was shared on when and how to carry out the protocol. They also ensured that the women properly followed the instructions and adhered to the study protocol. The staff then placed the saliva samples in the freezers. Sample collection occurred during the workweek, since more caregiving staff was available for monitoring the process. This timetable has been shown in previous studies to be the most appropriate [17].

After analyzing the samples, some had to be discarded because they had been collected more than 15 min after getting up and due to missing samples. One participant was removed from the clinical sample group, resulting in 9 saliva tests per woman and a total of 441 saliva samples (216 clinical samples and 225 non-clinical samples).

The same protocol was followed as in the clinical sample (women victims of IPV) for collecting information and extracting the saliva samples.

### 2.3. Instruments

#### 2.3.1. Salivary Cortisol

The radioimmunoassay (RIA) method of the Orion Diagnostica Spectria Cortisol Ria test [28] was used on saliva to quantitatively measure in vitro the concentrations of cortisol in humans. The saliva samples obtained from the 50 women were analyzed in laboratories at the Department of Psychology—Division of Biology of the University of Stockholm (Sweden).

#### 2.3.2. Personal Interview Protocol: Data Collection Model for the Victims of IPV

A personal interview protocol containing 376 fields was used for each woman in the clinical and non-clinical samples to obtain comprehensive and organized information regarding the different personal variables (in relation to personal, socio-economic, social/family information, social contexts, habits, motivations and social participation). For the clinical sample only, information was also collected on the variables related to the stressor, including type of abuse experienced, the acts committed during the abuse, coercive means used, reported complaints, the victim-reported person relationship (alleged abuser), the times the women had to leave home, possible medical attention, data about the alleged abuser, psychological situation (psychosomatic abuse), assistance variables (legal–psychological–social assistance).

The interview protocol was developed after researching many of the pre-existing models for interviews, together with information gathered about women with symptoms associated with IPV used in other areas such as law enforcement and the judicial system. In addition, other sources were reviewed such as the Protection Order Request Form of the Ministry of the Interior of Spain, based on a protocol of action by the Security Forces and Corps and coordination with the Judicial Bodies for the Protection of Victims of Domestic and Gender-based Violence (06-28-05), along with data from a semi-structured interview for victims of IPV [29].

#### 2.3.3. Alexithymia

The Toronto Alexithymia Scale, TAS-20 (Toronto Alexitimia Scale) is a reliable measuring instrument for the assessment of alexithymia and has therefore been applied to a diverse range of fields. In the present study, the Spanish-adapted version of the scale carried out by Martínez-Sánchez in 1996 was used. The scale consists of 20 items, each of which is answered by a Likert scale of five points (1: totally disagree; 5: totally agree) according to the degree of conformity and/or disagreement with each statement. The scale currently has three dimensions for its application: (1) difficulty identifying feelings and differentiating them from the bodily or physiological sensations that accompany emotional activation, (DIF); (2) difficulty describing feelings to others (DDF); and (3) externally-oriented thinking (EOT). All this makes the development and validation of this scale able to provide considerable support for the validity of the construct of alexithymia.

#### 2.3.4. State-Trait Anxiety

The Spanish-adapted version of the State–Trait Anxiety Inventory (STAI) Questionnaire was created by Nicolás Seisdedos Cubero in The STAI questionnaire consists of two self-assessment scales that measure two independent concepts of anxiety such as state (S) and trait (T). The questionnaire consists of 20 items related to trait anxiety and another 20 related to state anxiety. Each set of items is evaluated using a scale of 0 to 3, where 0 represents nothing or almost never and 4 represents much or almost always, respectively. The state anxiety (S/A) (STAI-S) is defined in a context of “transient emotional state or condition of the human organism characterized by subjective feelings”, while trait anxiety (T/A) (STAI-T) is characterized by a “relatively stable anxious propensity for which subjects differ in their tendency to perceive situations as threatening”.

#### 2.3.5. Post-Traumatic Stress Disorder Symptom Scale

The Post-Traumatic Stress Disorder Symptom Severity Scale (EGS) is a hetero- assessment scale designed as a structured interview, which serves to evaluate the symptoms and intensity of the post-traumatic stress disorder (PTSD) in victims of different traumatic events and who are sensitive to therapeutic change, according to the diagnostic criteria outlined by the DSM-IV. The scale consists of three subscales which are re-experiencing, avoidance and hyperarousal. The scale consists of 17 items, 5 of which refer to symptoms of re-experiencing, 7 to symptoms of avoidance and 5 to the symptoms of hyperarousal. The range is from 0 to 51 in the overall scale, from 0 to 15 in the re-experiencing subscale, from 0 to 21 in the avoidance subscale and from 0 to 15 in the hyperarousal subscale. This scale is structured in a Likert type format from 0 to 3, according to the frequency and intensity of the symptoms.

#### 2.3.6. Depression

Beck’s depression inventory (BDI) consists of 21 items and measures the intensity of symptoms of depression, especially with respect to cognitive disorders. In the questionnaire, several affirmation groups appear. The participant must choose among the statements that best describe his/her feelings during the past week, as well as the day the test is taken.

#### 2.3.7. Self-Esteem

The Rosenberg Self-Esteem Scale (SES) aims to assess the feeling of satisfaction that a person has with him or herself. It consists of 10 general items that score from 1 (strongly agree) to 4 (strongly disagree) on a Likert-type scale, which is useful to evaluate the level that abuse interferes in the self-esteem of the victim.

#### 2.3.8. Statistical Analysis

The statistical analysis of the data was performed using the SPSS software version 20 for Windows (United States, New York, IBM Corporation).

In order to establish any differences between the two populations of women (women victims and non-victims of IPV) with respect to psychometric parameters, the Kolmogorov–Smirnov test was previously used to determine the normality of the samples. The Student’s t test was applied once the normal distribution of the samples was confirmed.

In order to establish differences in CAR between the two populations of women (women victims and non-victims of IPV), and to determine that there were no differences between the different repetitions (three days of sampling), a double classification analysis of the variance (ANOVA) was used (two-way or bidirectional). The Fisher’s Test was used to assess the differences between the populations of non-victims and victims of IPV women who had higher levels of cortisol than those recorded in the CIRCORT database for the three collection time points.

## 3. Results

The psychological tests applied, and their subsequent statistical analysis is reflected in Table 1 The results show how the population of battered women differs significantly from the population of non-battered women with respect to the psychological variables under study.

A descriptive statistical summary of the cortisol data obtained from the samples is shown in Table 2.

When performing the bidirectional analysis of variance (ANOVA), applied to the CAR data determined by the area under the curve with respect to the increase (AUCi), it was observed that there are no differences between the data corresponding to each day of sampling—*F* (2, 141) = 0.33, *p* = 0.720—as well as between the CAR data of the two populations of women (women victims and non-victims of IPV) *F* (1, 141) = 1.690, *p* = 0.196 (Table 3).

The different cortisol concentrations of the three sampling collection times—Supplementary Tables S1–S3 were analyzed, since no differences were observed in the levels of CAR (AUCi) between the two populations of women (women victims and non-victims of IPV). This was achieved by determining the number of women from the two populations that had percentiles exceeding those values included in the CIRCORT global cortisol database. The Fisher test was applied to the values obtained to determine if there was a difference between the number of women who exceeded the cortisol percentiles included in the CIRCORT database, between the two populations (women victims and non-victims of IPV) in any of the sample collection time points. This revealed a highly significant difference in the three sampling days, where more women victims of IPV exceeded the cortisol levels of the CIRCORT database for 660mim (S3) as compared to the non-victims of IPV women sample (Table 4).

## 4. Discussion

The high case diversity of women victims of IPV necessarily leads to a high diversity in the different cortisol levels they present [15,16], making a clear diagnosis of their health situation from this physiological parameter difficult. For this reason, having standardized the different cortisol data in a global database (CIRCORT) offers us the opportunity to compare the different cortisol levels of women victims of IPV with these standardized values, with the aim of finding differences despite their diversity.

In the same way, if we wish to adequately represent the casuistic diversity with which we work, we need a study population that reflects this diversity as a priority over the total sample volume, a situation that our sample does present, which, although small in volume, is wide in casuistic diversity, which makes any result that can be attributed to the group in general significant.

Due to this diversity of casuistry among female victims of IPV, which leads to the high diversity in cortisol levels already mentioned; some studies have reported significant differences between the concentrations of salivary cortisol in the early hours of the morning in populations of women victims of IPV with respect to control populations [2,25] and high cortisol levels throughout the day [30]. However, our results show that similar levels of cortisol can be found in both populations during the first moments of the morning [26] as well as in the samples taken in the afternoon.

Conversely, the number of cases where these cortisol concentrations are elevated above the average parameters established by the CIRCORT global database () is significantly more abundant in the population of women victims of IPV with respect to the control in the samples taken in the afternoon. These results are consistent with other experiments that only compare with a control sample—and not with globally standardized levels as provided by the CIRCORT database, which show elevated levels of general cortisol during the course of the day, arriving at 11 h of sampling in women victims of IPV with a prolonged PTSD over time [30]. Another study comparing women victims of IPV experiencing PTSD to a non-PTSD-IPV control sample shows similar results where high levels of cortisol were observed at bedtime, while cortisol levels in the morning remained low [31]. The results of these studies are also similar to those of other studies related to pregnant women victims of IPV, where a high increase in salivary cortisol was detected in the afternoon, in close correlation with the physical and sexual violence suffered, as well as with scarce social resources and the perception of continued stress, as compared to the control sample [32]. Finally, another study which the main objective “*was to examine whether women’s violent reactions to IPV would significantly predict salivary cortisol, […] demonstrated that, psychological confrontation strategies predicted vespertine cortisol levels*” [7].

Other researchers have highlighted that due to the sleep difficulties that adults with PTSD and other types of disorders, such as those presented by female victims of IPV, and the relationship of the circadian rhythm with the cortisol level in the morning, it can be considered that taking measurements of cortisol levels in the afternoon will facilitate the detection of differences between samples [33]. Our results on the different cortisol levels in female IPV victims, for the first time, were obtained against globally standardized values, unify the different partial findings and establishing the analysis of afternoon salivary cortisol levels—no CAR—in comparison with standardized values from the global CIRCORT database as a suitable diagnostic tool for IPV consequences.

With regard to elements that have not been contemplated in this work, which might help to explain our results, we find studies on women showing that high levels of cortisol can be found in the afternoon, coinciding with a cerebral reduction of the hippocampal volume [34]. Other findings should be considered such as those on the genetic predisposition of the HPA reactivity to stress, with the actual cortisol levels obtained in the populations under study [35], or those that prove that abused women living in shelters showed higher CAR compared with women living with an abusive partner [36]. These lines of research should be considered in the future.

Although groups were carefully diagnosed, reliable instruments used and the well-controlled saliva sampling procedures, including three days of measurements and all this, contrasted by the use of the CIRCORT database, it was observed that the reduced size of groups, despite the high diversity achieved, decreased power to the results such that it would be desirable to investigate larger populations of women in future research.

## 5. Conclusions

Salivary cortisol concentration samples taken in the evening were significantly higher than those standardized in the CIRCORT database, from the women victims of IPV as compared to the control group. This difference could be determined due to the standardization of the different values of cortisol levels provided by the CIRCORT database, evidencing its usefulness as a diagnostic tool for problems as relevant as those triggered by IPV.

## Figures and Tables

**Table 1:** Psychological differences between the population of battered women and the population of non-battered women.

	Non-Victims of IPV Women (*N* = 25)			Women Victims of IPV (*N* = 24)			KS		T-Student
	*M*	*SD*	*SE*	*M*	*SD*	*SE*	*Z*	*p*	*p*
Depression (BDI)	9.14	7.019	0.993	16.58	11.475	1.623	1.407	0.038	0.0000 **
Total PTSD (EGS)	6.78	12.823	1.813	24.44	9.729	1.376	2.073	0.000	0.0000 **
Anxiety Trait (STAI-T)	23.86	11.210	1.585	35.46	12.362	1.748	0.483	0.974	0.0000 **
Anxiety State (STAI-S)	17.58	10.854	1.535	26.18	16.362	2.314	1.242	0.091	0.0015 **
Alexithymia (TAS-20)	49.18	14.337	2.208	54.30	13.007	1.839	0.808	0.531	0.0320 **
Self-esteem scale (S.E.S)	32.46	5.426	0.767	29.50	5.175	0.732	0.610	0.851	0.0030 **

Note. KS: Kolmogorov–Smirnov test; *M*: mean; *SD*: standard deviation; *Z*: Kolmogorov–Smirnov Z value; *p*: *p*-value. **: significant for α = 0.05. Intimate Partner Violence (IPV); Beck’s depression inventory (BDI); post-traumatic stress disorder (PTSD); Post-Traumatic Stress Disorder Symptom Severity Scale (EGS); Trait Anxiety (STAI-T); State Anxiety (STAI-S); Toronto Alexithymia Scale (TAS-20).

**Table 2:** Statistical descriptive summary of the different cortisol concentrations obtained in the samples collected from the two sample populations under study. Data expressed nmol/l.

	Non-Victims of IPV Women (*N* = 25)						Women Victims of IPV (*N* = 24)					
	1st Day		2nd Day		3rd Day		1st Day		2nd Day		3rd Day	
	*M*	*SD*	*M*	*SD*	*M*	*SD*	*M*	*SD*	*M*	*SD*	*M*	*SD*
CAR (AUCi)	2.136	3.660	2.192	3.460	1.252	4.280	3.156	5.760	2.481	4.300	2.665	3.500
Sample 1	11.788	6.733	9.983	4.833	11.567	6.040	12.852	6.579	12.852	6.706	12.600	6.381
Sample 2	18.100	11.896	14.946	9.245	16.896	6.279	17.124	8.053	17.236	8.073	15.078	8.195
Sample 3	4.325	2.374	4.558	2.240	4.467	3.142	5.796	7.772	5.508	4.204	4.961	2.844

Note. *M*: mean; *SD*: standard deviation; AUCi: area under the curve with respect to increase; CAR: Cortisol Awakening Response.

**Table 3:** Summary table of the ANOVA applied to the CAR data obtained.

Source	SS	df	MS	F	*p*
Variables: Non-victims of IPV women–Women victims of IPV	30.240	1	30.240	1.690	0.196
Repetitions: Days	11.790	2	5.900	0.330	0.720
Variables x Repetitions	7.960	2	3.980	0.220	0.803
Error	2516.640	141	17.850		
TOTAL	2566.630	146			

Note. SS: sum of squares; df: degrees of freedom; MS: mean squares; F: F de Snedecor; *p*: *p* value.

**Table 4:** The number of women with saliva cortisol levels within or outside the maximum percentiles as compared to the CIRCORT database, according to the sample collection and day.

	Non-Victims of IPV Women (*N* = 25)		Women Victims of IPV (*N* = 24)		
	In	Out	In	Out	*p* ^†^
Sample 1 1st day	23	2	22	2	1.000
Sample 1 2nd day	25	0	21	3	0.110
Sample 1 3rd day	23	2	23	1	1.000
Sample 2 1st day	21	4	19	5	0.725
Sample 2 2nd day	23	2	18	6	0.138
Sample 2 3rd day	23	2	22	2	1.000
Sample 3 1st day	19	6	8	16	0.004 *
Sample 3 2nd day	15	10	3	21	0.001 **
Sample 3 3rd day	19	6	4	20	0.000 **

Note. In: number of women with saliva cortisol levels within the maximum percentiles; Out: number of women with saliva cortisol levels outside. The maximum percentiles; ^†^: Fisher’s exact test. Significant for α = 0.01, *p* < 0.01 *; *p* < 0.001 **.

## Data Availability

Data available upon request due to ethical and privacy restrictions.

## Supplementary Materials

The following are available online at https://www.mdpi.com/article/10.3390/ijerph182010819/s1, Table S1: Statistical descriptive summary of the different cortisol concentrations obtained in the samples collected from the two sample populations under study. Data expressed nmol/l; Table S2: Summary table of the ANOVA applied to the CAR data obtained; Table S3: The number of women with saliva cortisol levels within or outside the maximum percentiles as compared to the CIRCORT database, according to the sample collection and day.

## References

[1] Breiding M.J., Basile K.C., Smith S.G., Black M.C., Mahendra R. (2015). Intimate Partner Violence Surveillance. Uniform Definitions and Recommended Data Elements.

[2] Yim I.S., Kofman Y.B. (2018). The psychobiology of stress and intimate partner violence. Psychoneuroendocrinology.

[3] Goldberg X., Espelt C., Porta-Casteràs D., Palao D., Nadal R., Armario A. (2021). Non-communicable diseases among women survivors of intimate partner violence: Critical review from a chronic stress framework. Neurosci. Biobehav. Rev..

[4] Heyman R.E., Kogan C.S., Foran H.M., Burns S.C., Slep A.M.S., Wojda A.K., Keeley J.W., Rebello T.J., Reed G.M. (2018). A case-controlled field study evaluating ICD-11 proposals for relational problems and intimate partner violence. Int. J. Clin. Health Psychol..

[5] Khoury J.E., Enlow M.B., Plamondon A., Lyons-Ruth K. (2019). The association between adversity and hair cortisol levels in humans: A meta-analysis. Psychoneuroendocrinology.

[6] Romero-Martínez A., Blasco-Ros C., Martínez M., Moya-Albiol L. (2019). Hormonal alterations in victimized women explained by their hostile reactions in coping with couple violence. Span. J. Psychol..

[7] Guyton A.C., Hall J.E. (2012). Compendio de Fisiología Médica. Hormonas Hipofisiarias y su Control por el Hipotálamo.

[8] Sanz-Martin A., Preciado-Mercado S., Olga-Inozemtseva G.L.I. (2019). Prefrontal dysfunction in girls with post-traumatic stress disorder secondary to child sexual abuse, and its relation to basal cortisol levels. J. Trauma Stress Disord. Treat..

[9] Lawrence E., Orengo-Aguayo R., Langer A., Brock R.L. (2012). The impact and consequences of partner abuse on partners. Partn. Abus..

[10] Morris M.C., Sanchez-Sáez F., Bailey B., Hellman N., Williams A., Schumacher J.A., Rao U. (2020). Predicting posttraumatic stress disorder among survivors of recent interpersonal violence. J. Interpers. Violence.

[11] Wilhelm I., Born J., Kudielka B.M., Schlotz W., Wüst S. (2007). Is the cortisol awakening rise a response to awakening?. Psychoneuroendocrinology.

[12] Hellhammer D.H., Wüst S., Kudielka B.M. (2009). Salivary cortisol as a biomarker in stress research. Psychoneuroendocrinology.

[13] Clow A., Hucklebridge F., Stalder T., Evans P., Thorn L. (2010). The cortisol awakening response: More than a measure of HPA axis function. Neurosci. Biobehav. Rev..

[14] Mikolajczak M., Quoidbach J., Vanootighem V., Lambert F., Lahaye M., Fillée C., de Timary P. (2010). Cortisol awakening response (CAR)’s flexibility leads to larger and more consistent associations with psychological factors than CAR magnitude. Psychoneuroendocrinology.

[15] Stalder T., Kirschbaum C., Kudielka B.M., Adam E.K., Pruessner J., Wüst S., Dockray S., Smyth N., Evans P., Hellhammer D.H. (2015). Assessment of the cortisol awakening response: Expert consensus guidelines. Psychoneuroendocrinology.

[16] Hellhammer J., Fries E., Schweisthal O., Schlotz W., Stone A., Hagemann D. (2007). Several daily measurements are necessary to reliably assess the cortisol rise after awakening: State- and trait components. Psychoneuroendocrinology.

[17] Ysseldyk R., Matheson K., Anisman H. (2017). Revenge is sour, but is forgiveness sweet? Psychological health and cortisol reactivity among women with experiences of abuse. J. Heal. Psychol..

[18] Alley J., Diamond L.M., Lipschitz D.L., Grewen K. (2020). Women’s cortisol stress responsivity, sexual arousability, and sexual history. Arch. Sex. Behav..

[19] D’Elia A.T.D., Juruena M.F., Coimbra B.M., Mello M.F., Mello A.F. (2021). Posttraumatic stress disorder (PTSD) and depression severity in sexually assaulted women: Hypothalamic-pituitary-adrenal (HPA) axis alterations. BMC Psychiatry.

[20] Jarnecke A.M., Barden E., Back S.E., Brady K.T., Flanagan J.C. (2018). Intimate partner violence moderates the association between oxytocin and reactivity to dyadic conflict among couples. Psychiatry Res..

[21] Garcia M.A., Junglen A., Ceroni T., Johnson D., Ciesla J., Delahanty D.L. (2020). The mediating impact of PTSD symptoms on cortisol awakening response in the context of intimate partner violence. Biol. Psychol..

[22] Liu S.Y., Wrosch C., Miller G.E., Pruessner J.C. (2014). Self-esteem change and diurnal cortisol secretion in older adulthood. Psychoneuroendocrinology.

[23] Pinto R.J., Correia-Santos P., Costa-Leite J., Levendosky A.A., Jongenelen I. (2016). Cortisol awakening response among women exposed to intimate partner violence. Psychoneuroendocrinology.

[24] Kim H.K., Tiberio S.S., Capaldi D.M., Shortt J.W., Squires E.C., Snodgrass J.J. (2014). Intimate partner violence and diurnal cortisol patterns in couples. Psychoneuroendocrinology.

[25] Miller R., Stalder T., Jarczok M., Almeida D.M., Badrick E., Bartels M., Boomsma D.I., Coe C.L., Dekker M.C., Donzella B. (2016). The CIRCORT database: Reference ranges and seasonal changes in diurnal salivary cortisol derived from a meta-dataset comprised of 15 field studies. Psychoneuroendocrinology.

[26] Stubbs A., Szoeke C. (2021). The effect of intimate partner violence on the physical health and health-related behaviors of women: A systematic review of the literature. Trauma Violence Abus..

[27] López-Ossorio J.J., Álvarez J.L.G., Pascual S.B., García L.F., Buela-Casal G. (2017). Risk factors related to intimate partner violence police recidivism in Spain. Int. J. Clin. Heal. Psychol..

[28] Hansen M., Garde A.H., Christensen J.M., Eller N.H., Netterstrøm B. (2003). Evaluation of a radioimmunoassay and establishment of a reference interval for salivary cortisol in healthy subjects in Denmark. Scand. J. Clin. Lab. Investig..

[29] Echeburúa E., Corral P., Zubizarreta B., Sarasua B. (1995). Trastorno de Estrés Postraumático Crónico en Víctimas de Agresiones Sexuales.

[30] Inslicht S.S., Marmar C.R., Neylan T.C., Metzler T.J., Hart S.L., Otte C., McCaslin S.E., Larkin G.L., Hyman K.B., Baum A. (2006). Increased cortisol in women with intimate partner violence-related posttraumatic stress disorder. Psychoneuroendocrinology.

[31] Cordero M.I., Moser D., Manini A., Suardi F., Sancho-Rossignol A., Torrisi R., Rossier M.F., Ansermet F., Dayer A., Rusconi-Serpa S. (2017). Effects of interpersonal violence-related post-traumatic stress disorder (PTSD) on mother and child diurnal cortisol rhythm and cortisol reactivity to a laboratory stressor involving separation. Horm. Behav..

[32] Valladares E., Peña R., Ellsberg M., Persson L., Högberg U. (2009). Neuroendocrine response to violence during pregnancy—Impact on duration of pregnancy and fetal growth. Acta Obstet. et Gynecol. Scand..

[33] Meewisse M.-L., Reitsma J.B., de Vries G.-J., Gersons B.P.R., Olff M. (2007). Cortisol and post-traumatic stress disorder in adults. Br. J. Psychiatry.

[34] Ruby E., Rothman K., Corcoran C., Goetz R.R., Malaspina D. (2015). Influence of early trauma on features of schizophrenia. Early Interv. Psychiatry.

[35] Edelman S., Shalev I., Uzefovsky F., Israel S., Knafo-Noam A., Kremer I., Mankuta D., Kaitz M., Ebstein R.P. (2012). Epigenetic and genetic factors predict women's salivary cortisol following a threat to the social self. PLoS ONE.

[36] Pinto R.J., Lamela D., Simães C., Levendosky A., Jongenelen I. (2019). Shelter versus living with abusive partner: Differences among mothers and children exposed to intimate partner violence. J. Child Fam. Stud..

