# Supplementary material for: Concentrations of Salivary Cortisol in Victims of Intimate Partner Violence According to the CIRCORT Database
Source: Int J Environ Res Public Health. 2021 Oct 14;18(20):10819. doi: 10.3390/ijerph182010819 (PMC8535720; doi:10.3390/ijerph182010819)
Supplement: Supplementary file 1 [file ijerph-18-10819-s001.zip › ijerph-1337356-supplementary.pdf]

Table S1. Statistical descriptive summary of the different cortisol concentrations obtained in the samples collected from the two sample populations under study. Data expressed nmol/l.

	**Non-victims of IPV women (*N* = 25)**						**Women victims of IPV (*N* = 24)**					
	1st day		2nd day		3rd day		1st day		2nd day		3rd day	
	M	SD	M	SD	M	SD	M	SD	M	SD	M	SD
CAR (AUCi)	2.136	3.660	2.192	3.460	1.252	4.280	3.156	5.760	2.481	4.300	2.665	3.500
Sample 1	11.788	6.733	9.983	4.833	11.567	6.040	12.852	6.579	12.852	6.706	12.600	6.381
Sample 2	18.100	11.896	14.946	9.245	16.896	6.279	17.124	8.053	17.236	8.073	15.078	8.195
Sample 3	4.325	2.374	4.558	2.240	4.467	3.142	5.796	7.772	5.508	4.204	4.961	2.844

Note. M: mean; SD: standard deviation; AUCi: area under the curve with respect to increase.

Table S2. Summary table of the ANOVA applied to the CAR data obtained.

**Source**	**SS**	**df**	**MS**	**F**	**p**
Variables: Non-victims of IPV women- Women victims of IPV	30.240	1	30.240	1.690	0.196
Repetitions: Days	11.790	2	5.900	0.330	0.720
Variables x Repetitions	7.960	2	3.980	0.220	0.803
Error	2516.640	141	17.850		
TOTAL	2566.630	146			

Note. SS: Sum of Squares; df: Degrees of freedom; MS: Mean Squares; F: F de Snedecor; p: p-value.

Table S3. The number of women with saliva cortisol levels within or outside the maximum percentiles as compared to the CIRCORT database, according to the sample collection and day.

	**Non-victims of IPV women (*N* = 25)**		**Women victims of IPV (*N* = 24)**		
	in	out	in	out	$p^{\dagger}$
Sample 1 1st day	23	2	22	2	1.000
Sample 1 2nd day	25	0	21	3	0.110
Sample 1 3rd day	23	2	23	1	1.000
Sample 2 1st day	21	4	19	5	0.725
Sample 2 2nd day	23	2	18	6	0.138
Sample 2 3rd day	23	2	22	2	1.000
Sample 3 1st day	19	6	8	16	0.004 **
Sample 3 2nd day	15	10	3	21	0.001 ***
Sample 3 3rd day	19	6	4	20	0.000 ***

Note. In: number of women with saliva cortisol levels within the maximum percentiles; Out: number of women with saliva cortisol levels outside the maximum percentiles; †: Fisher's exact test. Significant for $\alpha = 0.01$ * $p < 0.05$ ** $p < 0.01$ *** $p < 0.001$
